# Assembly and comparative analysis of the initial complete mitochondrial genome of *Primulina hunanensis* (Gesneriaceae): a cave-dwelling endangered plant

**DOI:** 10.1186/s12864-024-10247-9

**Published:** 2024-04-01

**Authors:** Lingling Chen, Xiang Dong, Hang Huang, Haixia Xu, Peninah Cheptoo Rono, Xiuzhen Cai, Guangwan Hu

**Affiliations:** 1 Department of Botany, College of Life Sciences, Hunan Normal University, Changsha, 410081 China; 2 CAS Key Laboratory of Plant Germplasm Enhancement and Specialty Agriculture, Wuhan Botanical Garden, Chinese Academy of Sciences, Wuhan, 430074 China; 3 Sino-Africa Joint Research Center, Chinese Academy of Sciences, Wuhan, 430074 China; 4 University of Chinese Academy of Sciences, Beijing, 100049 China; 5 Hubei Jiangxia Laboratory, Wuhan, 430200 China

**Keywords:** *Primulina hunanensis*, Mitochondrial genome, Organelle genome, Phylogenetic analysis

## Abstract

**Background:**

*Primulina hunanensis*, a troglobitic plant within the *Primulina* genus of Gesneriaceae family, exhibits robust resilience to arid conditions and holds great horticultural potential as an ornamental plant. The work of chloroplast genome (cpDNA) has been recently accomplished, however, the mitochondrial genome (mtDNA) that is crucial for plant evolution has not been reported.

**Results:**

In this study, we sequenced and assembled the *P. hunanensis* complete mtDNA, and elucidated its evolutionary and phylogenetic relationships. The assembled mtDNA spans 575,242 bp with 43.54% GC content, encompassing 60 genes, including 37 protein-coding genes (PCGs), 20 tRNA genes, and 3 rRNA genes. Notably, high number of repetitive sequences in the mtDNA and substantial sequence translocation from chloroplasts to mitochondria were observed. To determine the evolutionary and taxonomic positioning of *P. hunanensis*, a phylogenetic tree was constructed using mitochondrial PCGs from *P. hunanensis* and 32 other taxa. Furthermore, an exploration of PCGs relative synonymous codon usage, identification of RNA editing events, and an investigation of collinearity with closely related species were conducted.

**Conclusions:**

This study reports the initial assembly and annotation of *P. hunanensis* mtDNA, contributing to the limited mtDNA repository for Gesneriaceae plants and advancing our understanding of their evolution for improved utilization and conservation.

**Supplementary Information:**

The online version contains supplementary material available at 10.1186/s12864-024-10247-9.

## Introduction

*Primulina hunanensis*, a perennial herbaceous member of the Gesneriaceae family, thrives in low-light, barren cave environments, and exhibits distinctive environmental adaptation characteristics [1]. Renowned for its large green leaves, vibrant flowers, and elegant plant structure, this species has huge potential as an ornamental plant. Moreover, its capacity for asexual reproduction through leaf cuttings adds significant value to horticultural cultivation [2]. Despite these attributes, the unique habitat of *P. hunanensis*, confined to a single county in Hunan Province, China, makes it a species of conservation concern due to its restricted distribution and small population size. Conducting genomic research on *P. hunanensis* holds profound practical significance, aiming to promote the effective utilization of its germplasm resources.

Mitochondria plays a crucial role in oxidative metabolism within eukaryotic cells, through energy synthesis and conversion across the organismal life processes. Besides energy provision, mitochondria are involved in diverse physiological activities, including cellular information transmission, regulation of cell division, differentiation, and apoptosis [3–5]. Aligned with endosymbiosis theory, the evolution of chloroplasts and mitochondria began from their ancestral existence as free-living prokaryotes to their current status as specialized organelles with distinct functionalities [6–8]. Unlike nuclear genome in the majority of seed plants that is derived from biparental contributions, chloroplasts and mitochondria genomes have an exclusive maternal inheritance pattern [9]. This uniparental mode reduces the difficulty of genetic research, and therefore, organelle genomes are widely used in phylogenetics and evolutionary biology [10].

The first mtDNA within terrestrial plant *Marchantia polymorpha* was sequenced in 1992 [11], and since then, mtDNAs of various plants have been extensively studied. MtDNAs have been sequenced in dicotyledonous plants like *Brassica napus* [12], *Arabidopsis thaliana* [13], and *Beta vulgaris* [14], as well as monocotyledonous plants like *Oryza sativa* [15]. In contrast to cpDNAs, plant mtDNAs exhibit complex structure, including branching linear, unilinear, cyclic, and a combination of cyclic and linear [16, 17]. Generally, the mtDNA size within angiosperms ranges from 200 Kb to 3 Mb, with considerable variations evident not only across distinct plant species but also within genus [18]. These variations primarily arise from exogenous DNA elements integration, as well as prevalent repetitive sequences [19].

While plant mtDNA demonstrates considerable size and structural variation, genes exhibit relative conservation. Cytochrome *c* oxidase genes, NADH dehydrogenase genes, and ATP synthase genes, are few among many genes present in the vast majority of plants [20]. Key characteristics of plant mtDNA include sparsely distributed genes, abundant noncoding sequences, a wide array of repetitive sequences, and extensive RNA editing [21]. These characteristics contribute to the instability of mtDNA structure, making assembly more challenging. Consequently, the sequencing of mtDNA lags behind that of cpDNAs due to its complexity [22]. Nevertheless, related reports on plant mtDNAs are expected to increase rapidly as sequencing and assembly technologies advance.

Within the family Gesneriaceae, mtDNA of only two species: *Boea hygrometrica* [23] and *Haberlea rhodopensis* [24], have been reported. In this study, we report the initial complete mtDNA of *P. hunanensis*, complementing the cpDNA sequence previously reported (https://www.ncbi.nlm.nih.gov/, ON456288.1). Our results contribute to the growth of the mitochondrial DNA database specific to the *Primulina* genus, providing crucial genetic data essential for the conservation and cultivation of species within the Gesneriaceae family.

## Result

### Characteristics of the mtDNA of *P. hunanensis*

**Fig. 1:**
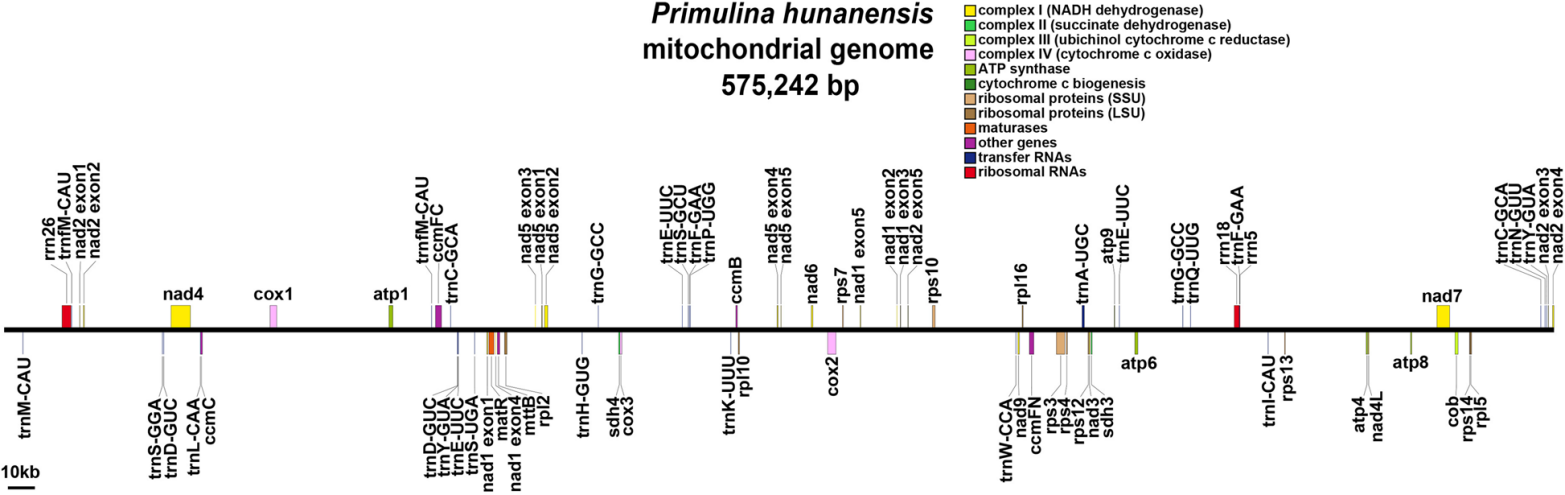
MtDNA map of *P. hunanensis*

Here, we obtained a computationally feasible mtDNA assembly of *P. hunanensis* and used a specific linear structure to represent the entire mtDNA sequence. It should be noted that the linear structure we present is one of the possible scenarios and is only used here as a representation. The total length of *P. hunanensis* mtDNA was 575,242 bp. and the GC content was 43.54% (Fig. 1). Annotation of the *P. hunanensis* mtDNA resulted in 37 PCGs, encompassing 24 mitochondrial core genes and 13 non-core genes. Among the core genes, there were one protein transport subunit gene (*mtt*B), nine NADH dehydrogenase genes (*nad*1, *nad*2, *nad*3, *nad*4, *nad*4L, *nad*5, *nad*6, *nad*7, and *nad*9), and one ubiquinol-cytochrome c reductase gene (*cob*). Additionally, there were three cytochrome c oxidase genes (*cox*1, *cox*2, and *cox*3), one maturase gene (*mat*R), and four cytochrome c biogenesis genes (*ccm*FC*, ccm*FN*, ccm*C*,* and *ccm*B), as well as five ATP synthase genes (*atp*1, *atp*4, *atp*6, *atp*8, and *atp*9). Non-core genes comprised four ribosomal large subunit genes (*rpl*2, *rpl*5, *rpl*10, *rpl*16), seven ribosomal small subunit genes (*rps*3, *rps*4, *rps*7, *rps*10, *rps*12, *rps*13, *rps*14), and two succinate dehydrogenase genes (*sdh*3, *sdh*4). A total of 20 tRNA genes were annotated, with seven tRNAs identified as multicopy, as determined by tRNAscan-SE. Additionally, three rRNA genes (*rrn*5, *rrn*18, *rrn*26) were present. Among them, 12 genes contain introns (Table 1).

**Table 1:** The mtDNA encoding genes of *P. hunanensis*

Group of genes	Name of genes
ATP synthase	*atp*1, *atp*4, *atp*6, *atp*8, *atp*9
NADH dehydrogenase	*nad*1^a^, *nad*2^a^, *nad*3, *nad*4^a^, *nad*4L, *nad*5^a^, *nad*6, *nad*7^a^, *nad*9
Cytochrome *b*	*cob*
Cytochrome *c* biogenesis	*ccm*B, *ccm*C, *ccm*FC^a^, *ccm*FN
Cytochrome *c* oxidase	*cox*1^a^, *cox*2^a^, *cox*3
Maturases	*mat*R
Protein transport subunit	*mtt*B
Ribosomal protein large subunit	*rpl*2, *rpl*5, *rpl*10, *rpl*16
Ribosomal protein small subunit	*rps*3^a^, *rps*4, *rps*7, *rps*10^a^, *rps*12, *rps*13, *rps*14
Succinate dehydrogenase	*sdh*3, *sdh*4
Ribosome RNA	*rrn*5, *rrn*18, *rrn*26
Transfer RNA	*trn*A-UGC^a^, *trn*C-GCA (× 2), *trn*D-GUC (× 2), *trn*E-UUC^a^ (× 3), *trn*F-GAA (× 2), *trn*fM-CAU (× 2), *trn*G-GCC (× 2), *trn*H-GUG, *trn*I-CAU, *trn*K-UUU, *trn*L-CAA, *trn*M-CAU, *trn*N-GUU, *trn*P-UGG, *trn*Q-UUG, *trn*S-GGA, *trn*S-GCU, *trn*S-UGA, *trn*W-CCA, *trn*Y-GUA (× 2)

The numerical value enclosed in parentheses denotes the gene’s copy number, exemplified by (× 2) to indicate the presence of two copies

^a^Labeled the genes that contain introns

**Fig. 2:**
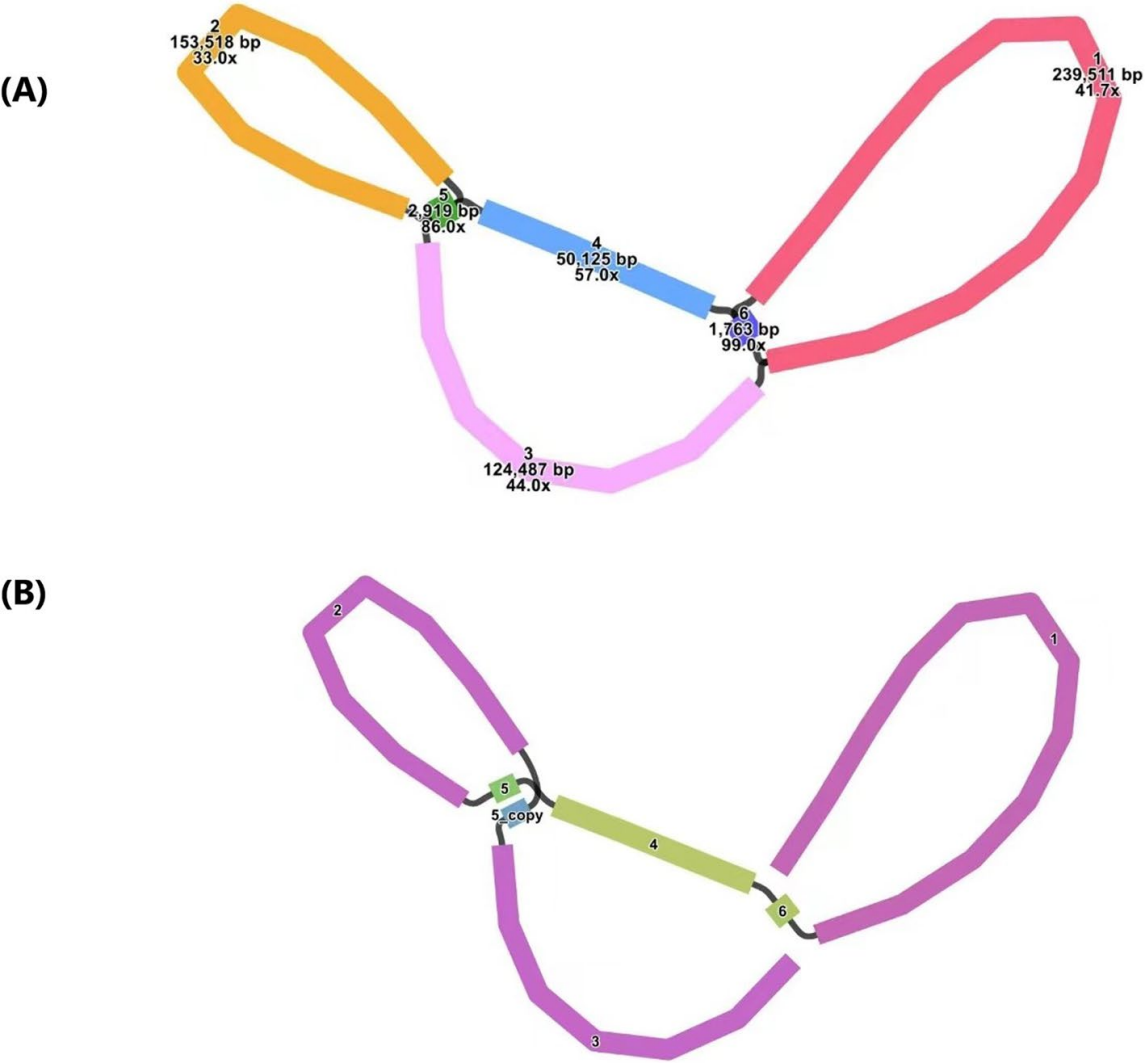
**A** Schematic of mtDNA of *P. hunanensis* based on Flye assembly (node ID labeled in the figure). **B** Unicycler hybrid assembly-based mtDNA of *P. hunanensis* (node ID labeled in the figure). The specific solution path is 3-5_copy-2–5-4–6-1

We used Bandage software to visualize the schematic of the mtDNA assembled based on long-reads data (Fig. 2A): the schematic contained six contigs, the naming, sequence length, and sequencing depth of contigs were shown in Table S1, and each contig represented a sequence obtained from the assembly, and if two contigs were connected by a black line, it means that there was an overlapping region between the two contigs. All these contigs constituted a complex multibranched closed structure that represented the complete mtDNA of the species. For several key nodes where branches exist, we used the Unicycler software to solve them on the basis of long-reads. The basic principle of Unicycler to solve duplicate regions is to map the relevant sequences at branching nodes onto long-reads, and when two sequences connected along the black line appear in the same long-read, it means that the long-read supports the connection of these two sequences. For branching nodes where there are multiple connections, those connections supported by more long-reads are preferred. The genome sequence of one line obtained after solving the branch nodes based on Unicycler was shown in Fig. 2B, with a specific solution path of 3-5_copy-2–5-4–6-1 (Table S2). Of course, we emphasize that the processing here is not the only form, because the structure of plant mtDNAs is not unique, but is in the midst of dynamic changes. This processing was chosen to facilitate the subsequent analysis.

### PCGs codon usage analysis

**Fig. 3:**
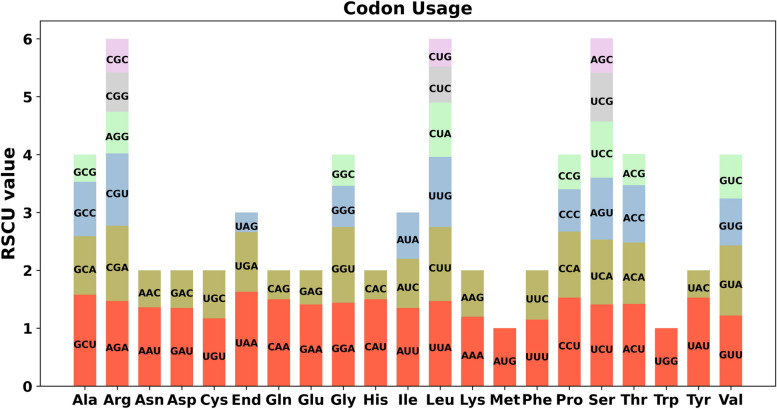
MtDNA relative synonymous codon usage of *P. hunanensis*. The x-axis represents codon families, with RSCU values representing specific codon frequency in comparison to uniform synonymous codon usage expected frequency

PCGs cumulative length in *P. hunanensis* amounted to 53,589 bp. Predominantly, to most PCGs, ATG was the initial codon, with the exception of ACG for *rps*10 and GTG for *rpl*16. While the termination codons for most PCGs were TAA, TGA, or TAG, the termination codon for *rps*10 was CGA, and for *atp*6, it was CAA (Table S3). The amino acids with greatest utilizing frequency were arginine (Arg), leucine (Leu), and serine (Ser), while methionine (Met) and tryptophan (Trp) had lower frequencies (Fig. 3).

The 64 codons were represented in PCGs, where RSCU values = 1 indicated no preference, and RSCU value > 1 denoted a preference for the amino acids. Excluding the start codon AUG and tryptophan (UGG), both with RSCU values of 1, a total of 31 codons were classified as high-frequency codons (RSCU > 1). Notably, termination codons displayed a preference for UAA, with highest RSCU value among mtPCGs at 1.63. Following closely was alanine (Ala) favoring GCU with RSCU value of 1.58. Interestingly, all NNA and NNT codons, excluding Leu (CUA, 0.92) and Ile (AUA, 0.8), exhibited RSCU values greater than or equal to 1.00 (Table S4).

### Repeat sequences analysis

**Fig. 4:**
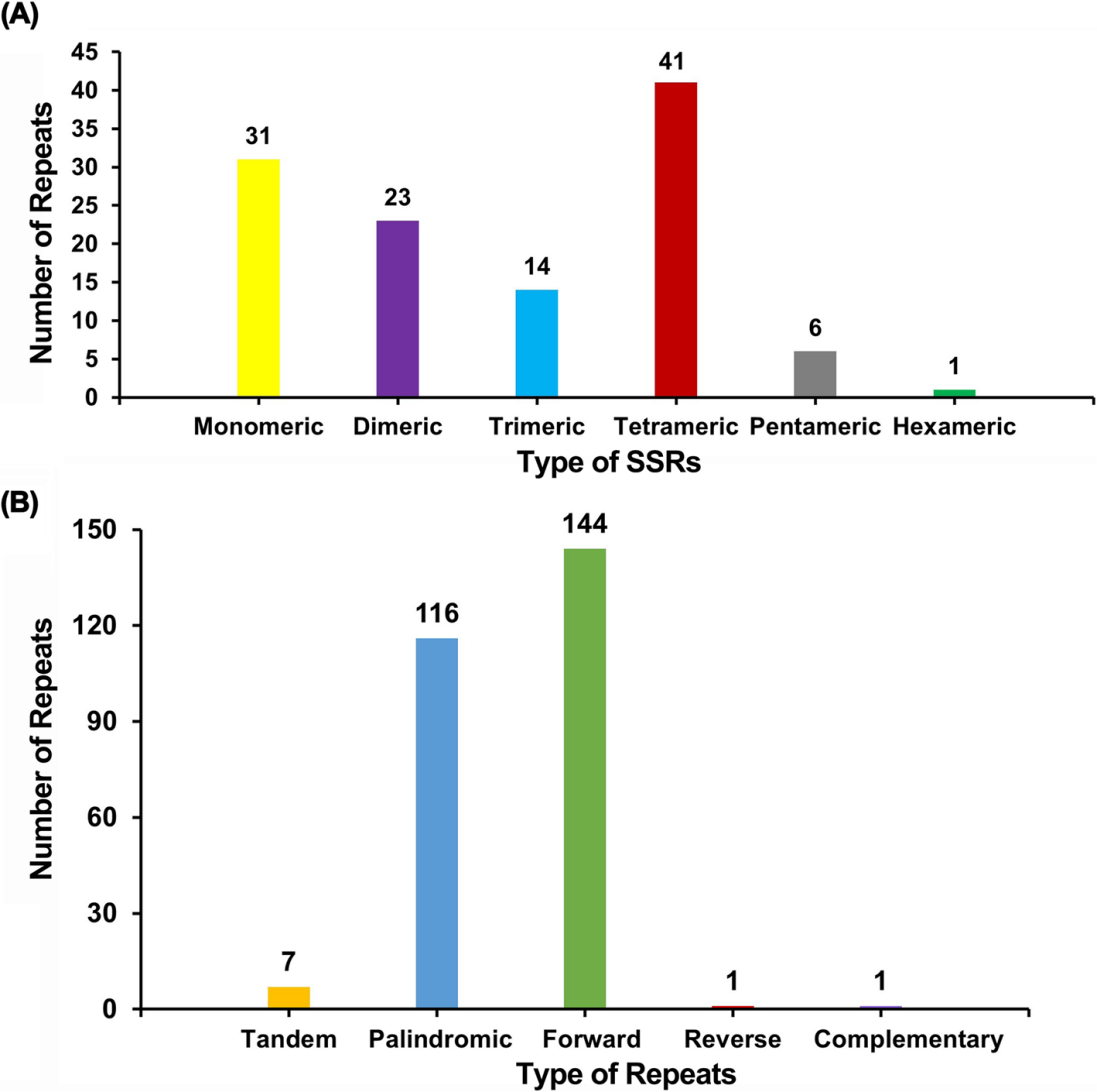
Histogram of repetitive sequence analysis. **A** The horizontal axis represents SSR types, while the vertical axis shows the number of repetitive fragments. The legend utilizes colors for clarity. **B** On the horizontal axis shows the types repeats, while the vertical axis illustrates the number of repeats. The colors distinguish the categories

Repetitive sequence analysis of the *P. hunanensis* mtDNA identified a total of 116 SSRs (Table S5), including 31 (26.72%) monomeric SSRs, 23 (19.83%) dimeric SSRs, 14 (12.07%) trimeric SSRs, tetrameric SSRs at 41 (35.34%) which constituted the highest number, pentameric SSRs 6 (5.17%), and hexameric SSRs 1 (0.87%) with the least number (Table S6) (Fig. 4A).

A total of seven tandem repeat sequences within the *P. hunanensis* mitochondrial DNA were identified, exhibiting over 71% similarity and lengths spanning 10–24 bp (Table S7). *P. hunanensis* mtDNA also harbored numerous dispersed repeats, comprising 262 pairs, with lengths ≥ 30 bp (Table S8), comprised 116 pairs of palindromic repeats (P), 144 pairs of forward repeats (F), one pair of reverse repeats (R), and one pair of complementary repeats (C) (Fig. 4B).

### Homologous analysis of genome sequences

**Fig. 5:**
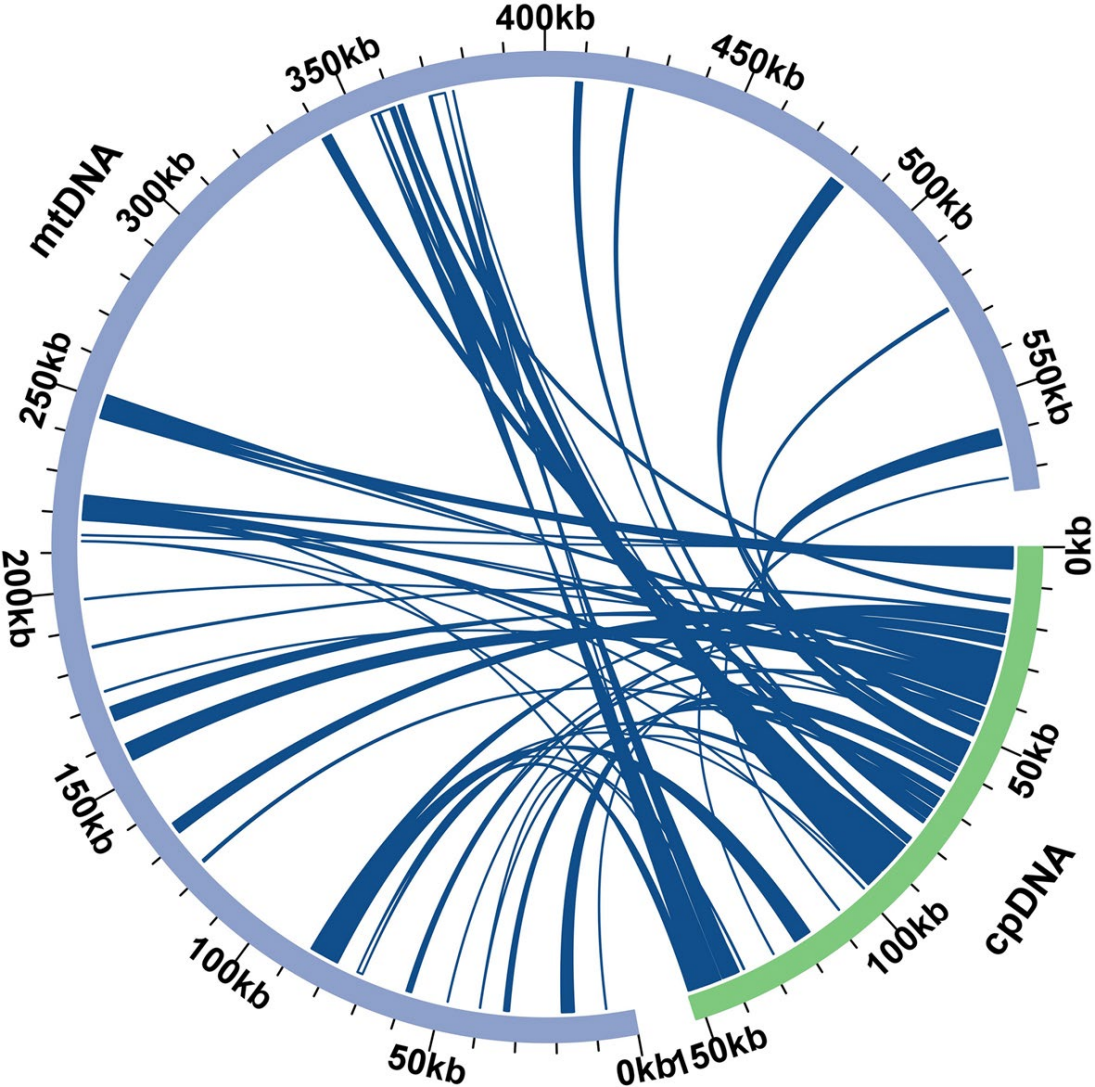
Homology analysis of the mitochondrial and chloroplast genomes of *P. hunanensis*. The grey arcs symbolize the mtDNA, the light green arcs denote the cpDNA, and blue lines connecting arcs signify homologous sequences

The cpDNA of *P. hunanensis* exhibited a length of 152,399 bp, encompassing 113 genes (Table S9). Through sequence similarity analysis, 65 homologous sequences with more than 80% similarity were identified between the cpDNA and mtDNA. These sequences varied in length from 41 to 4,443 bp, with mitochondrial plastid DNAs (MTPT) 32 emerging as the longest among them. Cumulatively, the homologous sequences length was 65,489 bp, constituting 11.38% of the overall mtDNA length (Fig. 5). Annotation of the homologous sequences resulted in a total of 33 complete genes comprising 22 PCGs (*acc*D, *mat*K, *ndh*A, *ndh*B, *ndh*H, *ndh*I, *ndh*J, *ndh*K, *pet*A, *pet*N, *psa*J, *psb*A, *psb*D, *psb*H, *psb*M, *psb*T, *rbc*L, *rpl*23, *rpl*33, *rps*14, *rps*18, *rps*7), and 11 tRNA genes (*trn*I-CAU, *trn*N-GUU, *trn*D-GUC, *trn*G-GCC, *trn*S-GCU, *trn*C-GCA, *trn*H-GUG, *trn*Y-GUA, *trn*E-UUC, *trn*M-CAU, *trn*fM-CAU). Additionally, some genes from chloroplasts were observed on these sequences, such as *ycf*2, *rpo*B, *psb*N, *psb*Z, *pet*B, *pet*D, etc., which are incomplete (Table S10).

### Phylogenetic analysis

**Fig. 6:**
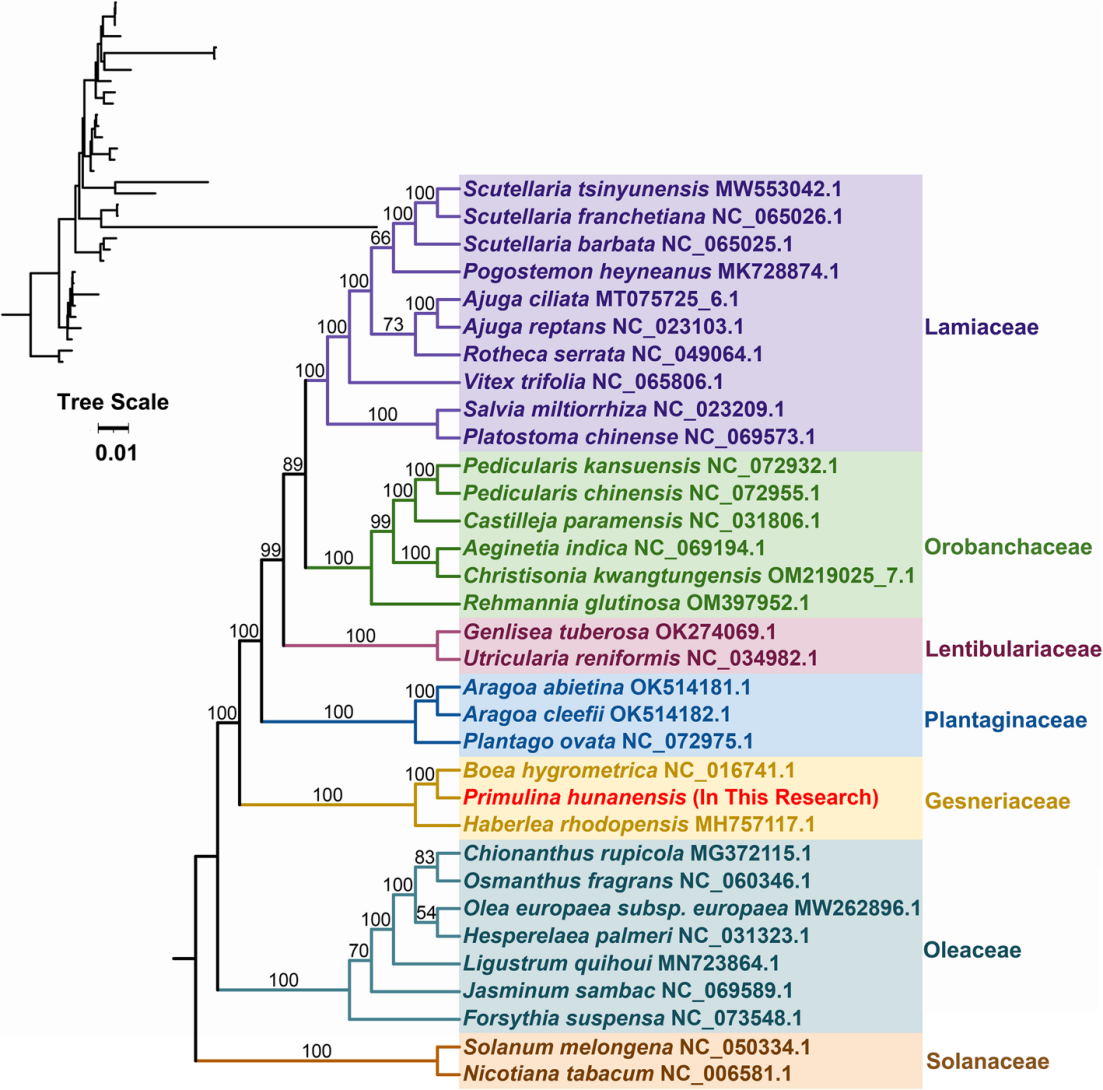
The phylogenetic associations of *P. hunanensis* alongside 32 other plant species. Phylogenetic tree constructed by maximum likelihood method based on 26 conserved mitochondrial genomic PCGs. *Solanum melongena* and *Nicotiana tabacum* were designated as outgroups. Numerical values at each node represent bootstrap probability. Different colors denote the respective families to which the species belong

Based on the DNA sequences of 26 conserved mtPCGs, a phylogenetic tree was constructed for 33 species under seven families of angiosperms. The selected common PCGs including *nad*2, *rpl*2, *ccm*C, *atp*6, *nad*3, *cox*3, *nad*4L, *rps*4, *rps*12, *atp*8, *rps*13, *atp*4, *ccm*B, *nad*4, *mat*R, *nad*1, *rps*3, *nad*5, *cox*2, *cob*, *ccm*FC, *ccm*FN, *nad*6, *mtt*B, *atp*1, *atp*9 were used to construct the phylogenetic tree. In the phylogenetic tree, 24 out of 30 branching nodes had bootstrap values of more than 90%, including 22 nodes with 100% bootstrap values. The topological arrangement of the phylogenetic tree, rooted in mtDNA sequences, aligned concordantly with latest identification by the Angiosperm Phylogeny Group (APG). Notably, *P. hunanensis* was classified within the family Gesneriaceae of the Lamiales order and demonstrated close phylogenetic relationship with *B. hygrometrica* (Fig. 6).

### RNA editing sites prediction

**Fig. 7:**
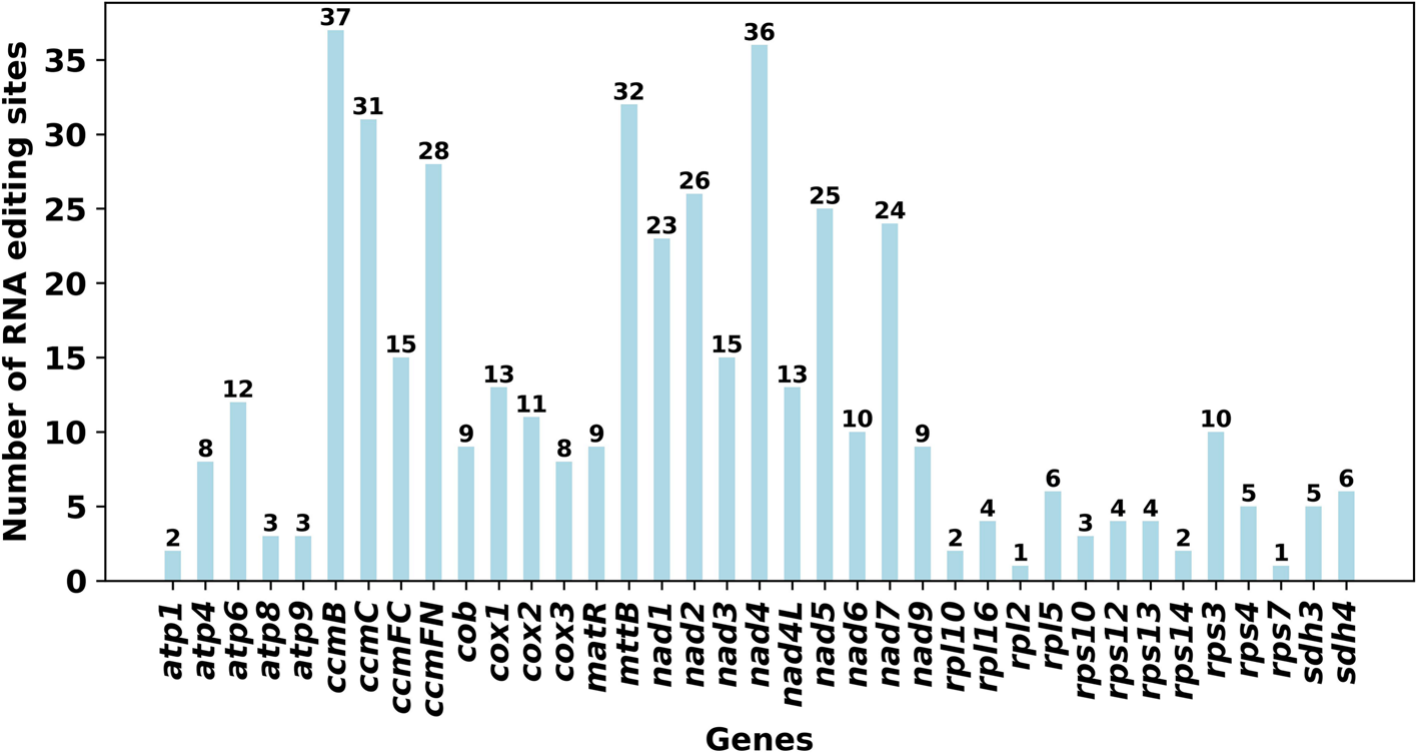
Illustration of RNA editing site distribution across individual mitochondrial PCGs in *P. hunanensis*. The blue bars depict the number of RNA-editing sites for each gene

Deepred-mt software predictions identified RNA editing events on 37 unique PCGs from *P. hunanensis* mtDNA. At a predictive performance of 0.9 (0 indicates no editing, while 1 represents editing. The closer the value is to 1, the higher the likelihood of editing occurrence), a total of 455 potential RNA editing sites were identified across the 37 mitochondrial PCGs, uniformly characterized by C to U conversions (Table S11). Notably, *ccm*B gene had the highest RNA editing sites frequency, totaling 37 edits. The *nad*4 gene displayed 36 RNA editing events, whereas both *rpl*2 and *rps*7 had only a single editing site (Fig. 7). A total of 43 codon variations were implicated in RNA editing sites, of which 45.94% of the amino acids had no alteration in hydrophobicity. On the other hand, 47.03% were predicted to undergo a transition from a hydrophilic to a hydrophobic state, while 6.59% were anticipated to shift from hydrophobic to hydrophilic (Table S12). Furthermore, RNA editing events were found to potentially introduce stop codons in the *atp*6 and *rps*10 genes.

### Collinearity analysis

**Fig. 8:**
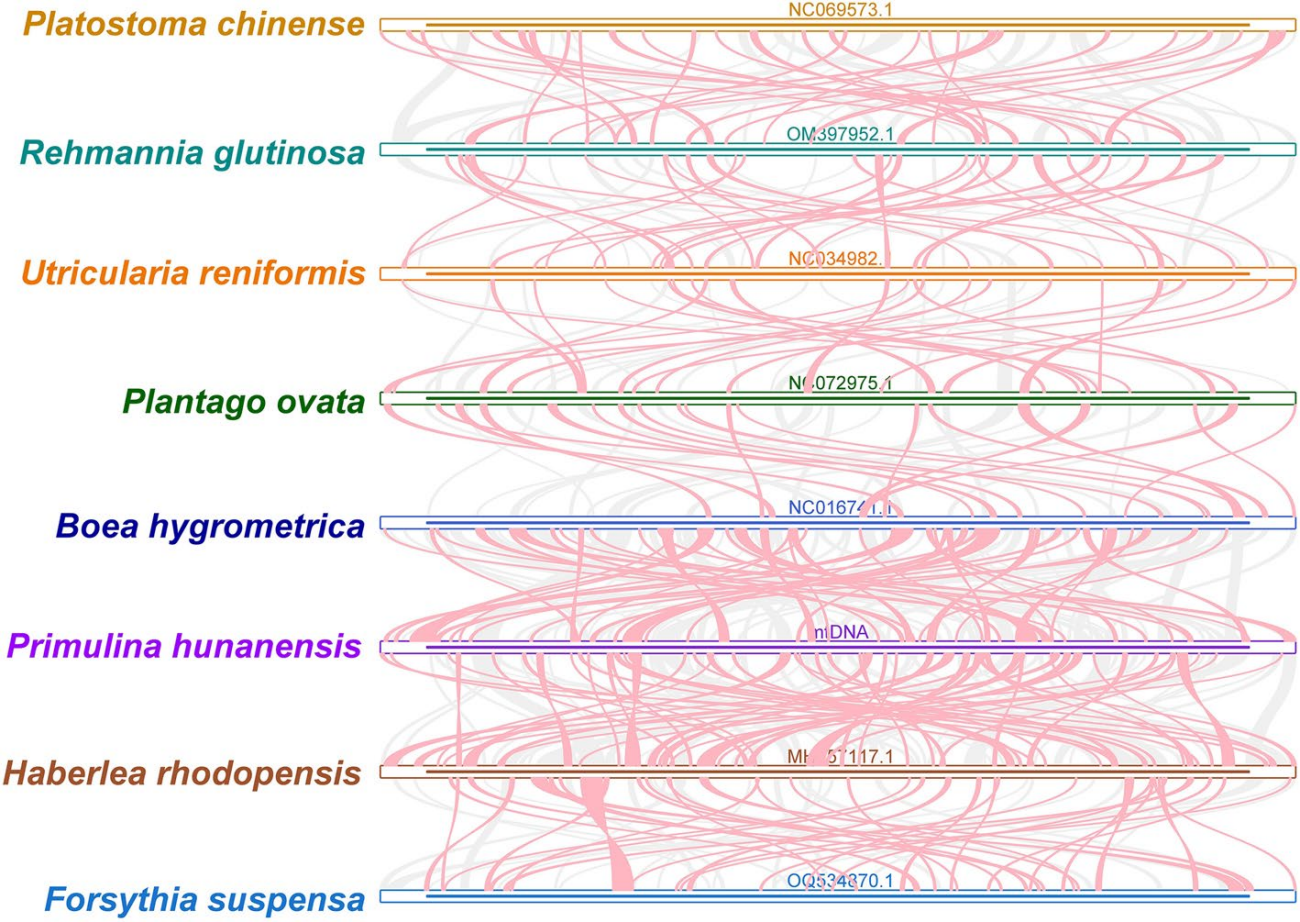
Collinearity analysis of *P. hunanensis*. The bar graph illustrates the mtDNA, with ribbons indicating homologous sequences between adjacent species. Red arcs signify inversion regions, gray areas denote regions of strong homology, and blank regions represent sequences unique to each species

The collinearity analysis, excluding collinear blocks with lengths < 0.5 kb revealed numerous homologous between *P. hunanensis* and related species within the order Lamiales. The largest collinear block was in comparison with *H. rhodopensis*, spanning approximately 10,037 kb, followed by *B. hygrometrica* with about 9,761 kb (Table S13). Additionally, there were unique blank regions in *P. hunanensis* sequences, exhibiting no homology to other species (Fig. 8). The results showed that the mtDNA alignments of the eight species of Labiatae were inconsistent, and that the mtDNA of *P. hunanensis* underwent extensive genomic rearrangements with its close relatives.

## Discussion

### Characteristics of the mtDNA of *P. hunanensis*

Currently, complete mtDNA have only been reported for *B. hygrometrica* and *H. rhodopensis* within the Gesneriaceae. In this study, we have assembled and determined that *P. hunanensis* mtDNA size was 575,242 bp, slightly longer than that of *B. hygrometrica* (510,519 bp) and *H. rhodopensis* (484,138 bp), with 43.54% GC content, similar to *B. hygrometrica* (43.27%) and *H. rhodopensis* (44.1%) [23, 24] Most land plants typically possess three rRNA genes [25], and we identified the same three rRNA genes (*rrn*18, *rrn*26, and *rrn*5) in the mtDNA of *P. hunanensis*.

The complexity and variations within plant mtDNA structure present persistent challenges for assembly and annotation efforts [21]. Unlike the circular structure presented by the mtDNAs of *B. hygrometrica* and *H. rhodopensis*, in this study, we chose to describe the mtDNA of *P. hunanensis* in terms of a linear structure supported by more long-reads, which does not imply that its actual structure is linear, but is more likely to exist in different conformations. Although most of the previous studies described the mtDNA structure in terms of a circular shape, the plant mtDNA is supposed to be assembled as a complex dynamic linear DNA collection of smaller circular and branched DNA molecules [26–28]. The results of this study further support the idea that multiple types of structures exist in the mtDNA of plants.

### Codon usage analysis of PCGs and repeat sequences

The PCGs in the mtDNA of *P. hunanensis* accounted for only 9.32% (53,589/575,242) of the total sequence length, potentially reflecting an accumulation of repetitive sequences during the evolutionary process [29]. In the mtDNA of organisms, ATG and GTG are common start codons, and TAA, TAG, and TGA are typical stop codons [30, 31]. Among the mitochondrial PCGs of *P. hunanensis*, the *rpl*16 gene has GTG as the start codon, and this phenomenon has also been widely reported in other plants such as maize [30]. In addition, ACG as the start codon of the *rps*10 gene, and CGA and CAA as the stop codons of the *rps*10 and *atp*6 genes, respectively, are thought to be the possible result of RNA editing modifications [32]. Analysis of codon usage through RSCU unveiled a notable adenine/thymine (A/T) richness at 3rd codon position within *P. hunanensis* mtDNA. The prevalence of NNA and NNT codons, with RSCU values equal to or surpassing 1.00, underscores a pronounced bias towards AT nucleotides at the third codon—a prevalent pattern observed in higher plants [33].

Repetitive elements, comprising simple repeats, tandem repeats, and dispersed repeats, are prevalent in plant mtDNAs and exert a significant influence on mitochondrial evolution [34, 35]. For SSRs, monomerics were dominated by A/T repeats, constituting 93.55% (29/31), while dimeric SSRs prominently featured AT/TA repeats, accounting for 56.52% (13/23). The high A/T composition in SSRs likely contributes to the overall AT richness observed in *P. hunanensis* mtDNAs. This aligns with broader trends seen in land plants, reflecting a consistent evolutionary trajectory within plant organelle genomes base composition [20, 33, 36]. The total length of the dispersed repeats was 13,887 bp, accounting for 2.41% (13,887/575,242) of the total mtDNA length. Predominantly, the dispersed repeats spanned 30–50 bp (216, 82.44%), with the longest palindromic repeat (P) measuring 147 bp, as well as the longest forward repeat (F) extending to 2,919 bp. These repetitive sequences not only contribute to mtDNA size expansion but also signify the frequent occurrence of intermolecular recombination in mtDNA [19, 25].

### Homology analysis

Angiosperms display extensive genetic material exchange between organelle genomes, through the transfer of DNA from the chloroplast to the mtDNA, known as MTPTs [37]. These fragments typically constitute 1–12% total mtDNA length. The mtDNA length of grape (*Vitis vinifera*) was 773,279 bp, with cpDNA fragments accounting for about 42% of the total, including 30 chloroplast PCGs and 17 tRNA genes [38]. Horizontal gene transfer is also documented between organelles and the nuclear genome [39, 40]. We found 65,489 bp of homologous sequences in the mtDNA and cpDNA of *P. hunanensis*, representing approximately 11.38% of the total mtDNA length. Annotation of these homologous sequences unveiled incomplete fragments originating from the cpDNA. Previous research indicates that, such homologous fragments vary among species in length and sequence [41]. Furthermore, genes migrating from chloroplasts to mitochondria often become pseudogenes over time, lacking functional significance, possibly due to sequence recombination [26, 42]. In this study, 11 intact tRNA genes, constituting 55% (11/20) of the total *P. hunanensis* mtRNA genes, were reported. The tRNA gene transference from chloroplasts to mitochondria is a recurring theme in plant evolution. Notably, as plants ascend from lower to higher taxa, the frequency of such tRNA transfers intensifies, aligning with the increasing demands for protein synthesis [43, 44]. The cpDNA of *P. hunanensis* contributes numerous sequences to the mitochondrial genome, enhancing the diversity of its mtDNA. Yet, the mechanisms driving sequence migration between the genomes and the expression of genes within migrated sequences remains unknown, warranting further investigation.

### RNA editing sites prediction

RNA editing involves alterations in amino acid composition during protein translation. This results in differences between the amino acids constituting the translated protein and the encoded information in the gene sequence, arising from nucleotide deletions, insertions, or substitutions in the transcribed mRNA molecule [45] Plant RNA editing predominantly takes the form of C-U conversions [46, 47]. Notably, RNA editing can induce changes in start and stop PCGs codons. For instance, *rps*10 exhibits an ACG start codon and a CGA stop codon, while *atp*6 has a CAA stop codon, alterations potentially attributed to RNA editing. Typically, new start and stop codons derived from RNA editing leads to the production of more evolutionarily conserved proteins, displaying high homology with corresponding proteins in other species. This conservation enhances the expression of mitochondrial genes [48]. Numerous RNA editing events culminate in amino acid changes, mostly converting hydrophilic amino acids to hydrophobic ones. In the mitochondria of *P. hunanensis*, 47.03% of amino acids in PCGs were predicted to undergo such changes following RNA editing. Hydrophobicity significantly influences protein folding, ensuring optimal protein conformation and functionality [49].

Earlier studies have reported 419 RNA editing sites in 35 PCGs of *H. rhodopensis*, 389 in 32 *B. hygrometrica* PCGs [24]. In this study, the 455 RNA editing sites identified were distributed across 37 PCGs within the mtDNA of *P. hunanensis*. RNA editing influences plant growth and development, constituting an essential feature of gene expression in plant mitochondrial genomes. Unedited RNA translation into protein typically results in severe or even lethal phenotypic outcomes [50, 51]. The exploration of RNA editing sites contributes to unraveling gene expression mechanisms within both chloroplasts and mitochondria, offering vital insights for predicting gene function [52, 53].

### Phylogenetic analysis and collinearity

The mtDNA has gained increased attention in phylogenetic analysis, especially with sequencing and assembly technologies rapid progression [33, 54]. In this study, *P. hunanensis*, *B. hygrometrica*, and *H. rhodopensis*, belonging to the Gesneriaceae family, formed a closely related cluster. Moreover, *P. hunanensis* displayed a closer phylogenetic relationship with *B. hygrometrica* compared to other relatives. It is worth noting that mitochondria, regarded as a relatively autonomous genetic system, might poses mitochondrial genes acquired from divergent plant species [55]. Consequently, constructing a phylogenetic tree solely based on mtDNA may not precisely mirror the accurate phylogenetic relationships [56, 57]. To this regard, further studies are needed incorporating the emerging understanding of mtDNA nature.

Homology analysis began in the 80s and prove crucial in elucidating species evolution [58–61]. Our current study unraveled numerous homologous collinear blocks shared between *P. hunanensis* and seven species within the order Lamiales. The observed disorder in the arrangement of these collinear blocks hints at extensive genomic rearrangements in the mtDNA of *P. hunanensis*, a phenomenon reported to be a potential driver of evolution and mtDNA diversity in *P. hunanensis* [62, 63].

## Conclusion

In this study, we successfully assembled and annotated the entire mtDNA of *P. hunanensis*, with a length of 575,242 bp and a GC content of 43.54%. A total of 60 genes, comprising 37 PCGs, 20 tRNA genes, and 3 rRNA genes were annotated. Our comprehensive analysis encompassed codon preference, repetitive sequences, genome homology, RNA editing events, phylogenetic relationships, and beyond. The data provided in this paper further support the idea that plant mtDNAs exist in multiple conformations, clarify the mitochondrial genomic features of *P. hunanensis*, and expand the mitochondrial genomic database of *Primulina* plant.

This research offers comprehensive insights into the organelle genetic characteristics and phylogenetic relationships of *P. hunanensis*, serving as an essential reference for subsequent investigations into the genomes of Gesneriaceae plants.

## Materials and methods

### Plant materials, DNA extraction and sequencing

Plant specimens used in this study were sourced from our curated collection of *P. hunanensis*, harvested in June 2022 from Jianghua Yao Autonomous County, Hunan Province, China. A corresponding voucher specimen (K. M. Liu and X. Z. Cai 31,330 (HNNU)) has been deposited in the Herbarium of Hunan Normal University.

Leaves collected from the natural habitats of the species, and from plants cultivated in the botanical gardens were dried using silica gel. Total DNA was extracted using a modified CTAB method [64], and evaluated for quality, quantity and purity using NanoDrop spectroscopy (Thermo Fisher Scientific) and 1% agarose gel electrophoresis. Library construction was conducted using the Truseq Nano DNA HT Sample Preparation Kit (Illumina USA) according to manufactures instructions. In brief, the DNA underwent sonication and fragmentation to 350 bp, with subsequent PCR amplification. Purified PCR products were obtained by the use of AMPure XP purification kit, while library's size distribution was estimated using Agilent 2100 Bioanalyzer. real-time PCR was used for quantification. Sequencing was performed using paired-end PE-150 bp on the Illumina HiSeq 2500 platform. Concurrently, the same DNA sample underwent single-molecule real-time sequencing using Nanopore-based ONT (Oxford Nanopore Technologies). This process yielded a total of 12.13 Gb of sequence reads, with 9.472 kb mean read length for sequencing and an N50 of 22.276 kb. Both the library construction and sequencing procedures were performed at Wuhan Benagen Technology Company.

### Assembly and annotation of mtDNA

Assembly of mtDNA: (1) The mtDNA of *P. hunanensis* was assembled using long-reads sequencing data. The assembly was performed using Flye software with default parameters [65], and graphical assembly results in GFA format were obtained. To identify contig fragments containing mtDNA, we built a database with the assembled contigs in FASTA format using makeblastdb [66]. The BLASTn algorithm was used, utilizing conserved mitochondrial genes from *Arabidopsis thaliana* as query sequences. The specific parameters used were "-evalue 1e-5, -outfmt 6, -max_hsps 10, -word_size 7, -task blastn-short" [67]. The resultant GFA files were visualized in Bandage software (v0.8.1) [68]. Subsequently, long-read data were aligned to graphical mtDNA fragments via BWA software (v0.7.17) [69]. The mitochondrial long-reads were selectively extracted to facilitate repetitive sequence regions within the plant’s mtDNA in-depth analysis.

(2) Simultaneously, the short-reads obtained from illumina were aligned to the mtDNA contigs acquired in step (1) through BWA software (v0.7.17) [69], resulting in the extraction of short-reads specific to mtDNA. The final assembly of mtDNA was performed using both the short-reads and long-reads in Unicycler [70].

For the mtDNA annotation, the PCGs were annotated in Geseq software (v2.03) [71] using *Arabidopsis thaliana* (NC_037304), *Liriodendron tulipifera* (NC_021152.1) as reference genomes. The tRNAs were annotated using tRNAscan-SE software (v.2.0.11) [72], while rRNAs were annotation in BLASTN software (v2.13.0) [67]. Genes with specific start and stop codons (*rpl*16, *rps*10, *atp*6, *rps*10) were checked and modified using Apollo software (v1.11.8) [73].

### Codon usage analysis

We extracted the mtDNA PCGs using Phylosuite software (v1.1.16) [74] and performed codon usage analysis in Mega software (v11.0) [75] through calculation of relative synonymous codon usage (RSCU) values.

### Analysis of repeated sequences

Simple sequence repeats (SSRs) were identified using Misa program (v2.1) [76]. Specifically, SSR motif size thresholds were configured at 10, 5, 4, 3, 3, 3 for 1–6 nucleotides. Tandem repeats were identified using Tandem repeats finder (v4.09) [77] with its default parameters. Detection of dispersed repeats was conducted using Reputer [78], comprising forward (F) / palindromic (P) / reverse (R) / complementary (C) repeats. Minimal repeat size was established at 30, with maximum computed repeats set to 5000, and the hamming distance maintained at 3.

### Homology analysis

The chloroplast DNA (cpDNA) assembly was performed using GetOrganelle software [79], followed by annotation in CPGAVAS2 [80]. Subsequent correction of the cpDNA annotation was done in CPGView [81]. Homologous fragments were detected using BLASTN software (v2.13.0) with parameters “-evalue 1e-5 -word_size 7 -outfmt 6, identity > 80%”, and the outcomes were visually represented utilizing the Circos package (v0.69.9) [82].

### Phylogenetic analysis

We retrieved mtDNAs from 32 species under seven families of angiosperms from the NCBI organelle resource database based on kinship (Table S14). A total of 26 conserved PCGs between these species were extracted using PhyloSuite software (v1.1.16) [74] and aligned in MAFFT software (v7.505) [83]. Using mtDNA from two Solanaceae species as outgroups, the phylogenetic analysis was performed based on the maximum likelihood method (ML) using IQ-TREE software (v1.6.12) with the parameters “–alrt 1000 -B 1000” [84], and visualized in ITOL software (v6) [85].

### RNA editing sites prediction

We predicted the RNA editing sites from all PCGs encoded within the mtDNA of *P. hunanensis*, specifically focusing on the conversion from cytosine (C) to uracil (U) using Deepred-mt tool [47]. All sites above a probability threshold of 0.9 were retained.

### Collinearity analysis

Pairwise comparisons of mtDNAs were conducted with seven closely related species in BLAST [67], with the following parameters: -evalue 1e-5 -word_size 7 -outfmt 6, identity > 80%, length > 500. A Multiple Synteny Plot, depicting the synteny relationships between *P. hunanensis* and its closely related counterparts was generated and visualized using MCscanX software [86].

### Supplementary Information

**Supplementary Material 1. **

## Data Availability

The complete mitochondrial genome of *Primulina hunanensis* is available at GenBank with accession numbers: OR840951.

## Abbreviations

cpDNA: Chloroplast genome
mtDNA: Mitochondrial genome
NCBI: National Center for Biotechnology Information
ONT: Oxford Nanopore Technologies
PCGs: Protein-coding genes
RSCU: Relative synonymous codon usage
SSRs: Simple sequence repeats
tRNA: Tranfer RNA
rRNA: Ribosomal RNA

## References

[1] Cai XZ, Tian J, Xiao SY, Peng L, Liu KM (2015). *Primulina Hunanensis* Sp Nov (Gesneriaceae) from a limestone area in southern Hunan, China. Nord J Bot.

[2] Li S, Cai X (2020). Studies on floral syndrome and breeding system of *Primulina Hunanensis*. Acta Horticulturae Sinica.

[3] Bonora M, De Marchi E, Patergnani S, Suski JM, Celsi F, Bononi A, Giorgi C, Marchi S, Rimessi A, Duszynski J (2014). Tumor necrosis factor-alpha impairs oligodendroglial differentiation through a mitochondria-dependent process. Cell Death Differ.

[4] Jorgensen C, Khoury M (2021). Musculoskeletal progenitor/stromal cell-derived mitochondria modulate cell differentiation and therapeutical function. Front Immunol.

[5] Madreiter-Sokolowski CT, Ramadani-Muja J, Ziomek G, Burgstaller S, Bischof H, Koshenov Z, Gottschalk B, Malli R, Graier WF (2019). Tracking intra- and inter-organelle signaling of mitochondria. FEBS J.

[6] Cavaliersmith T (1975). The origin of nuclei and of eukaryotic cells. Nature.

[7] Martin WF, Garg S, Zimorski V (2015). Endosymbiotic theories for eukaryote origin. Philos Trans R Soc B-Biol Sci.

[8] Zimorski V, Ku C, Martin WF, Gould SB (2014). Endosymbiotic theory for organelle origins. Curr Opin Microbiol.

[9] Birky CW (2001). The inheritance of genes in mitochondria and chloroplasts: laws, mechanisms, and models. Annu Rev Genet.

[10] Wallace DC, Singh G, Lott MT, Hodge JA, Schurr TG, Lezza AMS, Elsas LJ, Nikoskelainen EK (1988). Mitochondrial DNA mutation associated with Leber’s hereditary optic neuropathy. Science.

[11] Oda K, Yamato K, Ohta E, Nakamura Y, Takemura M, Nozato N, Akashi K, Kanegae T, Ogura Y, Kohchi T (1992). Gene organization deduced from the complete sequence of liverwort *Marchantia polymorpha* mitochondrial DNA. A primitive form of plant mitochondrial genome. J Mol Biol.

[12] Handa H (2003). The complete nucleotide sequence and RNA editing content of the mitochondrial genome of rapeseed (*Brassica napus* L.): comparative analysis of the mitochondrial genomes of rapeseed and *Arabidopsis thaliana*. Nucleic Acids Res.

[13] Unseld M, Marienfeld JR, Brandt P, Brennicke A (1997). The mitochondrial genome of *Arabidopsis thaliana* contains 57 genes in 366,924 nucleotides. Nat Genet.

[14] Kubo T, Nishizawa S, Sugawara A, Itchoda N, Estiati A, Mikami T (2000). The complete nucleotide sequence of the mitochondrial genome of sugar beet (*Beta vulgaris* L.) reveals a novel gene for tRNA(Cys)(GCA). Nucleic Acids Res.

[15] Notsu Y, Masood S, Nishikawa T, Kubo N, Akiduki G, Nakazono M, Hirai A, Kadowaki K (2002). The complete sequence of the rice (*Oryza sativa* L.) mitochondrial genome: frequent DNA sequence acquisition and loss during the evolution of flowering plants. Mol Genet Genomics.

[16] Clifton SW, Minx P, Fauron CMR, Gibson M, Allen JO, Sun H, Thompson M, Barbazuk WB, Kanuganti S, Tayloe C (2004). Sequence and comparative analysis of the maize NB mitochondrial genome. Plant Physiol.

[17] Kozik A, Rowan BA, Lavelle D, Berke L, Schranz ME, Michelmore RW, Christensen AC (2019). The alternative reality of plant mitochondrial DNA: one ring does not rule them all. PLoS Genet.

[18] Sloan DB, Alverson AJ, Chuckalovcak JP, Wu M, McCauley DE, Palmer JD, Taylor DR (2012). Rapid evolution of enormous, multichromosomal genomes in flowering plant mitochondria with exceptionally high mutation rates. PLoS Biol.

[19] Wynn EL, Christensen AC (2019). Repeats of unusual size in plant mitochondrial genomes: identification, incidence and evolution. G3-Genes. Genomes Genet.

[20] Bi C, Lu N, Xu Y, He C, Lu Z (2020). Characterization and analysis of the mitochondrial genome of common bean (*Phaseolus vulgaris*) by comparative genomic approaches. Int J Mol Sci.

[21] Gualberto JM, Mileshina D, Wallet C, Niazi AK, Weber-Lotfi F, Dietrich A (2014). The plant mitochondrial genome: dynamics and maintenance. Biochimie.

[22] Zardoya R (2020). Recent advances in understanding mitochondrial genome diversity. F1000Res.

[23] Zhang T, Zhang X, Hu S, Yu J (2011). An efficient procedure for plant organellar genome assembly, based on whole genome data from the 454 GS FLX sequencing platform. Plant Methods.

[24] Baev V, Ivanova Z, Yahubyan G, Toneva V, Apostolova E, Minkov G, Minkov I (2021). Analysis of the complete mitochondrial genome sequence of the resurrection plant *Haberlea rhodopensis*. Acta Biochim Pol.

[25] Cheng Y, He X, Priyadarshani SVGN, Wang Y, Ye L, Shi C, Ye K, Zhou Q, Luo Z, Deng F (2021). Assembly and comparative analysis of the complete mitochondrial genome of *Suaeda Glauca*. BMC Genomics.

[26] Anderson BM, Krause K, Petersen G (2021). Mitochondrial genomes of two parasitic *Cuscuta* species lack clear evidence of horizontal gene transfer and retain unusually fragmented ccmF(C) genes. BMC Genomics.

[27] He T, Ding X, Zhang H, Li Y, Chen L, Wang T, Yang L, Nie Z, Song Q, Gai J (2021). Comparative analysis of mitochondrial genomes of soybean cytoplasmic male-sterile lines and their maintainer lines. Funct Integr Genomics.

[28] Li J, Li J, Ma Y, Kou L, Wei J, Wang W (2022). The complete mitochondrial genome of okra (*Abelmoschus esculentus*): using nanopore long reads to investigate gene transfer from chloroplast genomes and rearrangements of mitochondrial DNA molecules. BMC Genomics.

[29] Gao H, Kong J (2005). Distribution characteristics and biological function of tandem repeat sequences in the genomes of different organisms. Zool Res.

[30] Bock H, Brennicke A, Schuster W (1994). Rps3 and rpl16 genes do not overlap in Oenothera mitochondria: GTG as a potential translation initiation codon in plant mitochondria?. Plant Mol Biol.

[31] Dong FG, Wilson KG, Makaroff CA (1998). The radish (*Raphanus sativus* L.) mitochondrial cox2 gene contains an ACG at the predicted translation initiation site. Curr Genet.

[32] Ichinose M, Sugita M (2016). RNA editing and its molecular mechanism in plant organelles. Genes (Basel).

[33] Yang H, Li W, Yu X, Zhang X, Zhang Z, Liu Y, Wang W, Tian X (2021). Insights into molecular structure, genome evolution and phylogenetic implication through mitochondrial genome sequence of *Gleditsia sinensis*. Sci Rep.

[34] Guo W, Zhu A, Fan W, Mower JP (2017). Complete mitochondrial genomes from the ferns *Ophioglossum californicum* and *Psilotum nudum* are highly repetitive with the largest organellar introns. New Phytol.

[35] Morley SA, Nielsen BL (2017). Plant mitochondrial DNA. Front Biosci.

[36] Wang X, Zhang R, Yun Q, Xu Y, Zhao G, Liu J, Shi S, Chen Z, Jia L (2021). Comprehensive analysis of complete mitochondrial genome of *Sapindus mukorossi* Gaertn.: an important industrial oil tree species in China. Ind Crop Prod.

[37] Wang X-C, Chen H, Yang D, Liu C (2018). Diversity of mitochondrial plastid DNAs (MTPTs) in seed plants. Mitochondrial DNA Part A.

[38] Goremykin VV, Salamini F, Velasco R, Viola R (2009). Mitochondrial DNA of *Vitis vinifera* and the issue of rampant horizontal gene transfer. Mol Biol Evol.

[39] Timmis JN, Ayliffe MA, Huang CY, Martin W (2004). Endosymbiotic gene transfer: organelle genomes forge eukaryotic chromosomes. Nat Rev Genet.

[40] Van Binh N, Vo Ngoc Linh G, Waminal NE, Park H-S, Kim N-H, Jang W, Lee J, Yang T-J (2020). Comprehensive comparative analysis of chloroplast genomes from seven *Panax* species and development of an authentication system based on species-unique single nucleotide polymorphism markers. J Ginseng Res.

[41] Choi K-S, Park S (2021). Complete plastid and mitochondrial genomes of *Aeginetia indica* reveal intracellular gene transfer (IGT), horizontal gene transfer (HGT), and cytoplasmic male sterility (CMS). Int J Mol Sci.

[42] Richardson AO, Rice DW, Young GJ, Alverson AJ, Palmer JD (2013). The "fossilized" mitochondrial genome of *Liriodendron tulipifera*: ancestral gene content and order, ancestral editing sites, and extraordinarily low mutation rate. BMC Biol.

[43] Bi C, Paterson AH, Wang X, Xu Y, Wu D, Qu Y, Jiang A, Ye Q, Ye N (2016). Analysis of the complete mitochondrial genome sequence of the diploid cotton *Gossypium raimondii* by comparative genomics approaches. Biomed Res Int.

[44] Qu Y, Zhou P, Tong C, Bi C, Xu L (2023). Assembly and analysis of the *Populus deltoides* mitochondrial genome: the first report of a multicircular mitochondrial conformation for the genus *Populus*. J Res.

[45] Brennicke A, Marchfelder A, Binder S (1999). RNA editing. Fems Microbiol Rev.

[46] Chen H, Deng L, Jiang Y, Lu P, Yu J (2011). RNA editing sites exist in protein-coding genes in the chloroplast genome of *Cycas taitungensis*. J Integr Plant Biol.

[47] Edera AA, Small I, Milone DH, Virginia Sanchez-Puerta M (2021). Deepred-Mt: Deep representation learning for predicting C-to-U RNA editing in plant mitochondria. Comput Biol Med.

[48] Galtier N (2011). The intriguing evolutionary dynamics of plant mitochondrial DNA. BMC Biol.

[49] Brenner WG, Mader M, Muller NA, Hoenicka H, Schroeder H, Zorn I, Fladung M, Kersten B (2019). High level of conservation of mitochondrial RNA editing sites among four *Populus* Species. G3 (Bethesda, Md).

[50] Schallenberg-Ruedinger M, Kindgren P, Zehrmann A, Small I, Knoop V (2013). A DYW-protein knockout in *Physcomitrella* affects two closely spaced mitochondrial editing sites and causes a severe developmental phenotype. Plant J.

[51] Toda T, Fujii S, Noguchi K, Kazama T, Toriyama K (2012). Rice MPR25 encodes a pentatricopeptide repeat protein and is essential for RNA editing of nad5 transcripts in mitochondria. Plant J.

[52] Hao W, Liu G, Wang W, Shen W, Zhao Y, Sun J, Yang Q, Zhang Y, Fan W, Pei S (2021). RNA editing and its roles in plant organelles. Front Genet.

[53] Li X-J, Zhang Y-F, Hou M, Sun F, Shen Y, Xiu Z-H, Wang X, Chen Z-L, Sun SSM, Small I (2014). Small kernel 1 encodes a pentatricopeptide repeat protein required for mitochondrial nad7 transcript editing and seed development in maize (*Zea mays*) and rice (*Oryza sativa*). Plant J.

[54] Choi I-S, Schwarz EN, Ruhlman TA, Khiyami MA, Sabir JSM, Hajarah NH, Sabir MJ, Rabah SO, Jansen RK (2019). Fluctuations in Fabaceae mitochondrial genome size and content are both ancient and recent. BMC Plant Biol.

[55] Liu D, Guo H, Zhu J, Qu K, Chen Y, Guo Y, Ding P, Yang H, Xu T, Jing Q (2022). Complex physical structure of complete mitochondrial genome of *Quercus acutissima* (Fagaceae): a significant energy plant. Genes.

[56] Liu L-X, Du Y-X, Folk RA, Wang S-Y, Soltis DE, Shang F-D, Li P (2020). Plastome evolution in Saxifragaceae and multiple plastid capture events involving *Heuchera* and *Tiarella*. Front Plant Sci.

[57] Yin H, Akimoto M, Kaewcheenchai R, Sotowa M, Ishii T, Ishikawa R (2015). Inconsistent diversities between nuclear and plastid genomes of AA genome species in the genus *Oryza*. Genes Genet Syst.

[58] Bonierbale MW, Plaisted RL, Tanksley SD (1988). RFLP maps based on a common set of clones reveal modes of chromosomal evolution in potato and tomato. Genetics.

[59] Lagercrantz U, Putterill J, Coupland G, Lydiate D (1996). Comparative mapping in *Arabidopsis* and *Brassica*, fine scale genome collinearity and congruence of genes controlling flowering time. Plant J.

[60] Tanksley SD, Bernatzky R, Lapitan NL, Prince JP (1988). Conservation of gene repertoire but not gene order in pepper and tomato. Proc Natl Acad Sci U S A.

[61] Wang X, Wang J, Jin D, Guo H, Lee T-H, Liu T, Paterson AH (2015). Genome alignment spanning major Poaceae lineages reveals heterogeneous evolutionary rates and alters inferred dates for key evolutionary events. Mol Plant.

[62] Liu B-B, Ren C, Kwak M, Hodel RGJ, Xu C, He J, Zhou W-B, Huang C-H, Ma H, Qian G-Z (2022). Phylogenomic conflict analyses in the apple genus *Malus* s.l. reveal widespread hybridization and allopolyploidy driving diversification, with insights into the complex biogeographic history in the Northern Hemisphere. J Integr Plant Biol.

[63] Yang J, Liu G, Zhao N, Chen S, Liu D, Ma W, Hu Z, Zhang M (2016). Comparative mitochondrial genome analysis reveals the evolutionary rearrangement mechanism in *Brassica*. Plant Biol.

[64] Porebski S, Bailey LG, Baum BR (1997). Modification of a CTAB DNA extraction protocol for plants containing high polysaccharide and polyphenol components. Plant Mol Biol Rep.

[65] Kolmogorov M, Yuan J, Lin Y, Pevzner PA (2019). Assembly of long, error-prone reads using repeat graphs. Nat Biotechnol.

[66] Cock PJ, Chilton JM, Grüning B, Johnson JE, Soranzo N (2015). NCBI BLAST + integrated into Galaxy. Gigascience.

[67] Chen Y, Ye W, Zhang Y, Xu Y (2015). High speed BLASTN: an accelerated MegaBLAST search tool. Nucleic Acids Res.

[68] Wick RR, Schultz MB, Zobel J, Holt KE (2015). Bandage: interactive visualization of de novo genome assemblies. Bioinformatics.

[69] Li H, Durbin R (2009). Fast and accurate short read alignment with Burrows-Wheeler transform. Bioinformatics.

[70] Wick RR, Judd LM, Gorrie CL, Holt KE, Unicycler (2017). Resolving bacterial genome assemblies from short and long sequencing reads. PLoS Comput Biol.

[71] Tillich M, Lehwark P, Pellizzer T, Ulbricht-Jones ES, Fischer A, Bock R, Greiner S (2017). GeSeq - versatile and accurate annotation of organelle genomes. Nucleic Acids Res.

[72] Lowe TM, Eddy SR (1997). tRNAscan-SE: a program for improved detection of transfer RNA genes in genomic sequence. Nucleic Acids Res.

[73] Lewis SE, Searle SMJ, Harris N, Gibson M, Lyer V, Richter J, Wiel C, Bayraktaroglu L, Birney E, Crosby MA (2002). Apollo: a sequence annotation editor. Genome Biol.

[74] Zhang D, Gao F, Jakovlic I, Zou H, Zhang J, Li WX, Wang GT (2020). PhyloSuite: an integrated and scalable desktop platform for streamlined molecular sequence data management and evolutionary phylogenetics studies. Mol Ecol Resour.

[75] Tamura K, Stecher G, Kumar S (2021). MEGA11: molecular evolutionary genetics analysis version 11. Mol Biol Evol.

[76] Beier S, Thiel T, Muench T, Scholz U, Mascher M (2017). MISA-web: a web server for microsatellite prediction. Bioinformatics.

[77] Benson G (1999). Tandem repeats finder: a program to analyze DNA sequences. Nucleic Acids Res.

[78] Kurtz S, Choudhuri JV, Ohlebusch E, Schleiermacher C, Stoye J, Giegerich R (2001). REPuter: the manifold applications of repeat analysis on a genomic scale. Nucleic Acids Res.

[79] Jin J-J, Yu W-B, Yang J-B, Song Y, dePamphilis CW, Yi T-S, Li D-Z (2020). GetOrganelle: a fast and versatile toolkit for accurate de novo assembly of organelle genomes. Genome Biol.

[80] Shi L, Chen H, Jiang M, Wang L, Wu X, Huang L, Liu C (2019). CPGAVAS2, an integrated plastome sequence annotator and analyzer. Nucleic Acids Res.

[81] Liu S, Ni Y, Li J, Zhang X, Yang H, Chen H, Liu C (2023). CPGView: a package for visualizing detailed chloroplast genome structures. Mol Ecol Resour.

[82] Zhang H, Meltzer P, Davis S (2013). RCircos: an R package for Circos 2D track plots. BMC Bioinformatics.

[83] Katoh K, Standley DM (2013). MAFFT multiple sequence alignment software version 7: improvements in performance and usability. Mol Biol Evol.

[84] Huelsenbeck JP, Ronquist F (2001). MRBAYES: bayesian inference of phylogenetic trees. Bioinformatics.

[85] Letunic I, Bork P (2019). Interactive tree of life (iTOL) v4: recent updates and new developments. Nucleic Acids Res.

[86] Wang Y, Tang H, DeBarry JD, Tan X, Li J, Wang X, Lee T-h, Jin H, Marler B, Guo H (2012). MCScanX: a toolkit for detection and evolutionary analysis of gene synteny and collinearity. Nucleic Acids Res.

